# Beyond Telomeres: Unveiling the Extratelomeric Functions of TERT in B-Cell Malignancies

**DOI:** 10.3390/cancers17071165

**Published:** 2025-03-30

**Authors:** Silvia Giunco, Maria Raffaella Petrara, Stefano Indraccolo, Vincenzo Ciminale, Anita De Rossi

**Affiliations:** 1 Section of Oncology and Immunology, Department of Surgery, Oncology and Gastroenterology, University of Padova, 35128 Padova, Italy; silvia.giunco@unipd.it (S.G.); stefano.indraccolo@unipd.it (S.I.); vincenzo.ciminale@unipd.it (V.C.); 2 Immunology and Diagnostic Molecular Oncology Unit, Veneto Institute of Oncology IOV-IRCCS, 35128 Padova, Italy; mariaraffaella.petrara@iov.veneto.it; 3 Basic and Translational Oncology Unit, Veneto Institute of Oncology IOV-IRCCS, 35128 Padova, Italy

**Keywords:** telomerase, TERT, TERT extratelomeric functions, B-cell malignancies, TERT-targeted therapy

## Abstract

The reactivation of telomerase enables cancer cells to maintain the telomere length, bypassing replicative senescence and achieving cellular immortality. In addition to its canonical role in telomere maintenance, accumulating evidence highlights telomere-length-independent functions of TERT, the catalytic subunit of telomerase. These extratelomeric functions involve the regulation of signaling pathways and transcriptional networks, creating feed-forward loops that promote cancer cell proliferation, resistance to apoptosis, and disease progression. This review explores the complex mechanisms by which TERT modulates key signaling pathways, such as NF-κB, AKT, and MYC, highlighting its role in driving autonomous cancer cell growth and resistance to therapy in B-cell malignancies. Furthermore, we discuss the therapeutic potential of targeting TERT’s extratelomeric functions. Unlike telomere-directed approaches, which may require prolonged treatment to achieve effective telomere erosion, inhibiting TERT’s extratelomeric functions offers the prospect of rapid tumor-specific effects. This strategy could complement existing chemotherapeutic regimens, providing an innovative and effective approach to managing B-cell malignancies.

## Simple Summary

Telomerase plays a critical role in maintaining the telomere length, thereby enabling tumor cells to escape senescence and acquire unlimited proliferative potential, a hallmark of cancer. Increasing evidence suggests that TERT, the catalytic subunit of telomerase, exerts additional biological functions that promote cancer progression independently of its role in telomere maintenance. These extratelomeric functions involve the regulation of signaling pathways that are critical for cell survival and proliferation, establishing feed-forward loops that drive cancer cell growth, resistance to apoptosis, and disease progression. This study underscores the importance of TERT’s non-canonical functions in B-cell malignancies and highlights the potential of targeting these functions as an innovative therapeutic strategy.

## 1. Introduction

A hallmark of cancer cells is their ability to evade replicative senescence, enabling continuous and unrestricted proliferation. In normal somatic cells, telomere shortening acts as a key barrier to cell proliferation. Telomeres, which are specialized DNA–protein complexes located at the ends of chromosomes, progressively shorten with each cell division. When telomeres reach a critical length, pathways leading to cellular senescence or apoptosis are activated, effectively preventing uncontrolled cell proliferation [1]. In the absence of this protective mechanism, cells may continue to divide, resulting in further telomere erosion, which promotes chromosomal instability, a key factor in cancer development.

In most cancers, the reactivation of telomerase, a specific protein–RNA complex containing an internal RNA component (telomerase RNA component, TERC) and a catalytic protein (telomerase reverse transcriptase, TERT) with telomere-specific reverse transcriptase activity, is essential for telomere maintenance, leading to limitless cell proliferation and tumor growth [1,2,3].

Beyond its canonical role in maintaining the telomere length, accumulating evidence indicates that TERT, the rate-limiting component of telomerase [4], is linked to several telomere-length-independent functions that contribute to tumor progression, including roles in cell growth, gene expression regulation, mitochondrial function, oxidative stress resistance, and apoptosis prevention [5,6].

Given telomerase’s central role in maintaining the telomere length and replicative capacity in cancer cells, it is considered an attractive therapeutic target. However, strategies solely aimed at targeting telomere maintenance may face limitations, such as the prolonged treatment required to achieve telomere attrition necessary for anti-tumor effects and the potential toxicity to normal tissues. The discovery of TERT’s extratelomeric functions in tumor growth and progression suggests that targeting these functions could offer therapeutic benefits beyond its direct effect on telomeres.

In this review, we provide a comprehensive overview of the extratelomeric functions of TERT in B-cell malignancies, emphasizing their therapeutic potential as novel targets for cancer treatment.

## 2. Telomere Erosion—A Driving Force in Oncogenesis

Telomeres are composed of repetitive hexanucleotide sequences (5′-TTAGGG-3′) located at the ends of each chromosome. Together with specialized telomeric DNA-binding proteins, e.g., the shelterin complex, they form a three-dimensional structure that provides chromosomal protection [7]. This structure prevents the recognition of the chromosomal termini as damaged DNA, thereby avoiding an inappropriate DNA damage response (DDR) and chromosomal rearrangements [8]. Therefore, telomeres are essential in maintaining the integrity and stability of DNA.

In most human somatic cells, each round of cell division results in the loss of 50–150 base pairs of telomeric DNA, due to DNA polymerase’s inability to fully replicate chromosome ends [9], and, after continuous population doublings, telomeres progressively shorten, eventually reaching a critical length that renders them dysfunctional. Critically short telomeres lose their ability to bind shelterin proteins, failing to repress the DDR. This dysfunction activates ATM and ATR checkpoint kinases, leading to the upregulation of TP53 [10] and, in specific contexts, the activation of the RB1/p16 signaling pathways [11].

Telomere erosion is a physiological process associated with aging and serves as a critical tumor-suppressive mechanism, providing an initial proliferative barrier to tumor formation. However, cells with defective checkpoint pathways, or mutations in TP53 and/or RB1, can bypass the senescence barrier, continuing to divide despite critically short telomeres. This unchecked replication exacerbates telomere dysfunction [12], leading to increased chromosomal instability, including end-to-end chromosome fusion and the rearrangement of chromosomes, which are key events in carcinogenesis [13]. Studies on hematological malignancies, especially chronic lymphocytic leukemia (CLL), have provided evidence that telomere shortening correlates with disease progression [14,15,16,17,18].

During malignant transformation, cancer cells with acquired tumor-promoting mutations and genomic instability must stabilize their telomeres to restore the protective capping function, bypass cellular crisis, and achieve unlimited proliferative potential. In most cancer cells (85–90%), telomere maintenance is achieved through telomerase reactivation [1,2]. In a smaller subset of cancers (10–15%), telomeres are maintained through a recombinant-based mechanism named Alternative Lengthening of Telomeres (ALT) [19].

## 3. Telomerase

Telomerase, encoded by the *TERT* gene, is a specialized reverse transcriptase enzyme that uses the template region of the internal RNA component (TERC) to maintain the telomere length by adding hexamer repeats to telomeres [20]. Although TERC is widely distributed across tissues, TERT acts as the rate-limiting component of the complex, and its levels generally correlate with telomerase activity [4]. Telomerase is active during embryonic development, sustaining a high rate of cell division, but it is absent in the majority of adult somatic cells due to the suppression of *TERT* transcription [21]. Minimal telomerase activity is maintained in specific tissues that undergo rapid division, such as adult stem cells like intestinal crypt cells [22], regenerating hepatocytes [23], activated B and T lymphocytes [24,25], and male germ cells, where the TERT levels are tightly regulated [26].

The downregulation of telomerase in somatic cells limits the number of cellular divisions, leading to progressive telomere shortening, a hallmark of the aging process. As mentioned, telomerase is inappropriately reactivated in the vast majority of cancers [1,2]. High levels of TERT and telomerase activity in cells harboring tumor-promoting mutations and genomic instability confer cellular immortality by preventing cellular replicative senescence and apoptosis induced by telomere erosion, thus promoting tumor formation and progression [27,28].

### Regulation of TERT Expression and Telomerase Activity

The levels of TERT are regulated through multiple mechanisms, including transcriptional, post-transcriptional, and epigenetic processes. The *TERT* promoter contains several binding sites for a variety of transcriptional repressors and activators. The main transcriptional activators include SP1, MYC, HIF1A, AP-2, members of the E-twenty-six (ETS) family and ternary complex factors (TCF), NF-κB, and β-catenin [29,30,31]. The activation of pathways such as NF-κB and β-catenin enhances TERT transcription, either through direct binding to the *TERT* promoter or indirectly by upregulating different TERT transcriptional activators, such as MYC. On the other hand, several factors, such as WT1, TP53, NFX-1, MAD1, and CTCF, act as repressors of the *TERT* promoter [29].

Additionally, somatic mutations in the *TERT* promoter are commonly linked to increased *TERT* expression. The specific mutations −124 C>T and −146 C>T enhance *TERT* expression by generating novel binding sites for ETS/TCF transcription factors [32,33]. These *TERT* promoter mutations are prevalent in many cancers [34] but are rare in hematological malignancies [35,36]. The *TERT* promoter also contains a cluster of CpG sites that contributes to transcriptional regulation through DNA methylation [29].

At the post-transcriptional level, several mechanisms can influence the *TERT* mRNA levels, including alternative splicing [37] and microRNA (miRNA) levels. MiRNAs can either directly target *TERT* mRNA or modulate the transcription factors involved in regulating *TERT* expression [38,39,40].

Telomerase activity is further influenced post-translationally through modifications such as the ubiquitylation and phosphorylation of the TERT subunit [41]. The phosphorylation of the TERT protein by the PI3K/AKT pathway promotes telomerase activity by facilitating its nuclear localization [42]. TERT undergoes nuclear–cytoplasmic shuttling, which is essential for its assembly and activity regulation [21,43]. Moreover, under basal conditions, 10–20% of TERT localizes to the mitochondria [44]. Under oxidative stress, TERT is reversibly excluded from the nucleus and accumulates in the mitochondria, where it performs telomere-length-independent functions [45].

The modulation of telomerase is also influenced by oncogenic viruses, including hepatitis B virus (HBV), hepatitis C virus (HCV), Kaposi’s sarcoma-associated herpes virus (KSHV), Epstein–Barr virus (EBV), cytomegalovirus (CMV), and human T-cell leukemia virus-1 (HTLV-1) [46,47,48,49]. Of interest, the infection of resting B cells in vitro by EBV can lead to the generation of immortalized, continuously proliferating lymphoblastoid cell lines (LCLs), which may serve an in vitro model of EBV-driven post-transplant lymphoproliferative disorders [50]. In LCLs and EBV-driven B-cell malignancies, viral latent membrane protein 1 (LMP1), the major EBV oncoprotein, promotes telomerase activity by enhancing *TERT* transcription through the NF-κB and MAPK/ERK1/2 pathways [51]. This mechanism ensures the unlimited proliferation and transformation of chronically EBV-infected cells, contributing to their oncogenic potential.

## 4. Telomeres and Telomerase in Normal and Neoplastic B Cells

Quiescent circulating B lymphocytes exhibit unique telomere and telomerase dynamics, characterized by longer telomeres and higher telomerase activity compared to other blood cells [52,53]. Germinal center (GC) B cells, which undergo clonal expansion, display significantly longer telomeres and elevated telomerase activity compared to naive or memory B cells, suggesting that telomerase actively elongates telomeres, preserving the proliferative capacity necessary for effective clonal expansion during a normal GC reaction [54]. However, the telomerase activity in GC B cells is significantly lower than that observed in neoplastic cells [24].

Increased telomerase activity has been observed in various B-cell malignancies, including diffuse large B-cell lymphoma (DLBCL), Burkitt lymphoma (BL), follicular lymphoma, mantle cell lymphoma, and CLL [55]. Telomerase activity is positively correlated with the proliferation index of tumor cells [55], and the direct relationship between telomerase activity and proliferation underscores its essential role in lymphomagenesis and leukemogenesis [55].

During lymphomagenesis, variations in the levels and timing of telomerase activation contribute to the differences in telomere lengths among various lymphoid tumors. Notably, BL exhibits the longest telomeres, followed by DLBCL and follicular lymphoma. Marginal zone lymphoma and multiple myeloma display intermediate telomere lengths, while CLL and mantle cell lymphoma exhibit the shortest telomeres [56,57]. Of interest, among the established BL cell lines, the EBV-positive ones show longer telomeres than the EBV-negative ones [58].

In Hodgkin lymphoma (HL), telomeric dysfunction may play a pivotal role in chromosomal instability [59] and in the transition from mononuclear Hodgkin cells to the multinucleated Reed–Sternberg (RS) cells, which are characterized by disrupted shelterin complexes and significant telomere shortening and represent the hallmark end-stage cells of HL [60]. The EBV oncoprotein LMP1 may exacerbate telomeric dysfunction by downregulating shelterin proteins [61]. In addition, during the progression to the RS phenotype, cells may activate the ALT mechanism, and both pathways for telomere maintenance may coexist in HL [62]. Telomere parameters, particularly the proportion of very short telomeres, may serve as predictive biomarkers for the therapy response [63].

Notably, in the in vitro system to generate LCL, during the early phases of the EBV-induced proliferation of primary B cells, the telomere lengths remain constant or even increase [64,65]. However, after prolonged in vitro culture, EBV-infected lymphocytes experience telomere shortening and genomic instability, mediated by the displacement of the shelterin protein TRF2 from telomeres by EBV [65]. In this context, only EBV-positive cells with sustained telomerase activity, driven by increased TERT levels promoted by the EBV oncoprotein LMP1, achieve unlimited cell proliferation, thereby promoting the evolution of malignant clones carrying aneuploidy and stabilized telomere lengths [51,66,67,68]. In contrast, telomerase-negative EBV-infected cells, despite their extended lifespan, ultimately undergo cellular senescence and reach the end of their replicative potential due to critical telomere shortening [66,67].

The role of telomerase activity in maintaining the telomere length makes it a valuable prognostic biomarker. In patients with CLL, the TERT levels are inversely correlated with the telomere length, and the telomere length/TERT level profile delineates subgroups of CLL with different clinical outcomes. Specifically, cases characterized by high TERT levels and short telomeres are independently associated with faster disease progression [14]. In B-acute lymphoblastic leukemia, a cancer that originates from a B-cell precursor lineage and is particularly common among pediatric patients, high TERT levels and telomerase activity correlate with poor clinical outcomes and lower survival rates [69,70,71,72]. Furthermore, high TERT levels and telomerase activity are associated with an unfavorable prognosis in multiple myeloma and mantle cell lymphoma [73].

## 5. TERT’s Non-Canonical Functions: Focus on B-Cell Malignancies

TERT is also associated with various telomere-length-independent functions that contribute to tumor development, including the enhancement of proliferation, resistance to apoptosis, and the promotion of inflammation, invasion, and metastasis [5,6,74].

Notably, TERT plays a pivotal role as a transcription (co-)factor, regulating gene expression and modulating key signaling pathways, such as WNT/β-catenin, NF-κB p65, and MYC, which drive cancer onset and progression [5]. Specifically, TERT binds to WNT-responsive promoters and acts as a transcriptional cofactor with BRG1, a chromatin remodeler, influencing WNT target gene expression [75]. Additionally, TERT interacts with the NF-κB p65 subunit, facilitating its recruitment to NF-κB-responsive promoters, including those for IL6 and TNFα, thereby directly regulating NF-κB-dependent gene expression [76]. TERT also plays a direct role in the MYC pathway by interacting with MYC at the protein level, promoting its stabilization and enhancing its binding to target gene promoters [77]. Accumulating evidence suggests the existence of feed-forward regulatory loops between TERT and these transcriptional factors [74,78]. In these loops, the transcription factors regulate *TERT* expression, while TERT, in turn, modulates their transcriptional levels and/or their cellular compartmentalization and stability [74,78]. Once activated, these loops contribute to tumor progression by influencing multiple hallmarks of cancer [6].

TERT also localizes within mitochondria, where it is imported via the interaction of its mitochondrial-targeting signal with translocases of the outer (TOM 20 and TOM 40) and inner (TIM 23) mitochondrial import machinery [44,79,80,81,82,83], where it binds to specific regions of mitochondrial DNA, including coding areas around the ND1 and ND2 genes of complex I, as well as the coding regions for ribosomal 12S and 16S RNAs, COX I and III, various tRNAs, and subunits 6 and 8 of ATP synthase [44,84]. Several studies have demonstrated that TERT lowers the mitochondrial and cellular reactive oxygen species (ROS) levels in various cell types under basal conditions and upon oxidative stress, ultimately protecting mitochondrial DNA from oxidative damage [44,83,85,86]. However, the mechanisms underlying these protective effects remain unclear, as some studies suggest a direct mitochondrial function of TERT [44], while others propose an indirect role through the modulation of nuclear-encoded antioxidant pathways [86,87], independent of its mitochondrial localization.

A comprehensive evaluation of whether the observed non-canonical roles of TERT are TERC-dependent remains lacking. However, the findings from some studies suggest that TERT’s extratelomeric functions do not require TERC. It has been demonstrated that, in mitochondria, where TERC has not been detected, TERT can perform reverse transcription by associating with other mitochondrial RNAs. Additionally, TERT retains its mitochondrial functions even when introduced into human cells lacking TERC [84]. Furthermore, ectopic TERT has been shown to enhance proliferation in TERC-negative cells [88].

In B-cell malignancies, TERT also exhibits extratelomeric functions that impact several pathways sustaining autonomous cancer cell proliferation, resistance to apoptosis, and tumor progression. A summary of studies exploring these functions in B-cell malignancies, primarily derived from short-term TERT inhibition approaches, is provided in Table 1.

Several studies reveal a strong association between increased TERT levels and the enhanced proliferation and viability of tumor B cells. Indeed, short-term TERT inhibition consistently exhibits anti-proliferative and pro-apoptotic effects in both in vitro and in vivo models, occurring within timeframes shorter than those required to trigger telomere damage induced by telomere attrition [77,78,89,91,92,94,96,97,98,99,100]. These effects are frequently linked to alterations in the NF-κB and MYC signaling pathways [76,77,78,94,95,96,97,99].

In B cells, NF-κB signaling is crucial for growth and survival, with its deregulation identified as a key factor for leukemic and lymphoma cell proliferation and viability [103,104]. MYC, a central regulator closely involved in driving uncontrolled proliferation in cancers, including hematological malignancies [105], is transcriptionally regulated by NF-κB via direct effects on the promoter of both wild-type and translocated *MYC* [106,107]. NF-κB can also activate the *TERT* promoter directly [108] or indirectly through MYC [109,110].

Recent evidence suggests the existence of a multifaceted regulatory loop between TERT, NF-κB p65, and MYC in B-cell malignancies that contributes to cancer progression [78]. The short-term inhibition of TERT by BIBR1532 (a chemical inhibitor of TERT, affecting its conformation and processivity) [111,112,113] in in vitro models of B-cell malignancies, such as LCLs and BL cell lines, impairs cell proliferation with the accumulation of cells in the S-phase and induces apoptosis associated with the activation of the DDR via a telomere-length-independent mechanism [91]. In fact, the length of telomeres remains unchanged between treated and untreated cells, and the DNA damage induced by the treatment is randomly distributed rather than specifically localized at telomeres [91]. TERT inhibition in these models downregulates NF-κB p65 nuclear localization, reducing its availability at target promoters. Consequently, the transcription of key NF-κB p65 target genes, such as MYC, BCL2, and Survivin, is diminished. Additionally, MYC downregulation compromises proliferation by increasing the p21 levels and promoting its nuclear localization [78]. In the nucleus, p21 inhibits DNA replication by interfering with PCNA-dependent DNA polymerase activity and CDK2-dependent replication origin firing, leadings to S-phase prolongation [114]. This S-phase extension suggests replication fork stalling, which activates the DDR pathway, essential for fork protection [115]. These findings support the concept that TERT acts as a transcriptional amplifier in cancer independently of its canonical role in telomere maintenance. Its inhibition might, therefore, serve as an effective strategy to counteract tumor growth.

Along with the anti-proliferative effect, short-term TERT inhibition can induce apoptosis mediated by the suppression of NF-κB-driven Survivin and/or BCL2 expression [78,94,97,98,99,100]. TERT inhibition may also trigger apoptosis through the inhibition of the AKT pathway [89,100]. In B-cell malignancy models, both short-hairpin (sh)RNA- and BIBR1532-mediated TERT inhibition result in the dephosphorylation of AKT1. Dephosphorylated AKT1 might activate FOXO3, leading to the upregulation of the pro-apoptotic protein NOXA, or promote the dephosphorylation/stabilization of BAD to induce cell death [89,100]. Given the critical role of AKT signaling in regulating B-cell proliferation and survival [116], further investigation is needed to elucidate the precise mechanisms by which TERT modulates AKT1 phosphorylation.

Consistent with its potential role in influencing the ROS levels, it has been recently demonstrated that short-term TERT inhibition via siRNA in an in vitro model of precursor-B acute lymphoblastic leukemia induces lipid ROS production, while reducing the total cellular antioxidant capacity. This effect is associated with the upregulation of ferroptosis promoters such as ACSL4 and the suppression of inhibitors such as SLC7A11 [98].

Furthermore, TERT plays an active role in the interaction between oncogenic viruses and host cells during neoplastic transformation [49,117,118], particularly in EBV-associated B-cell malignancies, where high levels of TERT support the tumorigenic program associated with EBV latency, characterized by the expression of the LMP1 gene [68]. TERT promotes this effect through the upregulation of NOTCH2 via the NF-κB signaling pathway. NOTCH2, in turn, regulates its target gene, BATF, a transcription factor that suppresses BZLF1, the master regulator of the EBV lytic cycle, thereby facilitating EBV latency [90]. Consistent with this mechanism, the siRNA- or shRNA-mediated silencing of TERT, or its inhibition through the chemical suppression of TERT transcription factors, promotes the EBV lytic cycle by upregulating BZLF1 [68,89,93]. By triggering the viral lytic cycle, TERT inhibition induces cell death and sensitizes EBV-infected cells to antiviral drugs [89], supporting the concept that TERT inhibition may be of therapeutic importance even in EBV-driven malignancies.

**Figure 1:**
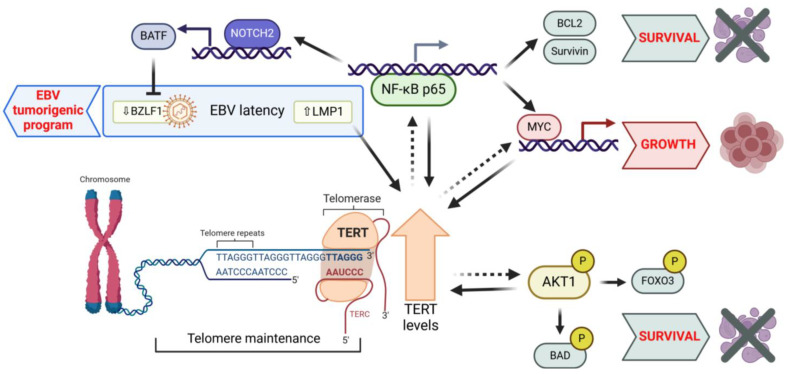
TERT’s canonical and non-canonical roles affecting proliferation and survival in B-cell malignancies. TERT activation sustains the replicative potential of both EBV-driven and virus-unrelated B-cell malignancies by maintaining the telomere length, thereby enabling unlimited proliferation. Beyond this canonical function, TERT interacts with key cancer-related pathways, including NF-κB, MYC, and AKT (dashed lines). These extratelomeric functions promote tumor progression by enhancing cell proliferation and survival, often within feed-forward regulatory loops in both EBV-driven and virus-unrelated B-cell malignancies. Additionally, TERT supports the tumorigenic EBV latency program in infected cells. TERT: telomerase reverse transcriptase; TERC: telomerase RNA component; NF-κB: RELA proto-oncogene, NF-kB subunit; MYC: MYC proto-oncogene, bHLH transcription factor, AKT1: AKT serine/threonine kinase 1; EBV: Epstein–Barr virus; BATF: basic leucine zipper ATF-like transcription factor; NOTCH2: notch receptor 2; BCL2: BCL2 apoptosis regulator; BAD: BCL2-associated agonist of cell death; FOXO3: forkhead box O3; BZLF1: BamHI Z fragment leftward open reading frame 1.

A schematic model illustrating TERT’s canonical and non-canonical roles affecting proliferation and survival in B-cell malignancies is presented in Figure 1.

## 6. Telomerase Inhibition Strategies

As TERT is highly expressed in the vast majority of cancer cells but largely absent in normal somatic cells, it represents an attractive and selective target for anti-cancer therapies. This specificity minimizes off-target effects while ensuring broad applicability across a wide range of tumor types.

Several strategies have been developed to target telomerase, including both direct and indirect telomerase inhibitors, suicide gene therapy, telomerase peptide and DNA vaccines, and TERT-based adoptive cell therapy, and several of these approaches have progressed to clinical trials [119,120,121,122].

Among these, Imetelstat (GRN163L) is the most advanced drug in clinical evaluation. This lipidated 13-mer thiophosphoramidate oligonucleotide competitively binds the TERC template region, inhibiting telomerase activity. By progressively shortening telomeres, it reduces cancer cell proliferation and induces cell death [119]. While Imetelstat faced limitations in solid tumor trials due to hematologic dose-limiting toxicities, including thrombocytopenia, lymphopenia, and neutropenia [123,124], it demonstrated significant efficacy in hematologic malignancies, particularly in myelodysplastic syndromes [125,126,127,128].

Another intriguing candidate is BIBR1532. BIBR1532 is a non-competitive non-nucleoside small molecule that selectively inhibits telomerase activity by binding to a hydrophobic pocket on the N-terminal domain (TEN) of TERT. This interaction disrupts telomerase assembly and stability by locking TERT in a closed conformation, affecting the enzyme’s active loop conformation and processivity [111,112,113]. Consequently, by inducing this structural constraint, BIBR1532 may also influence TERT’s non-canonical functions. Preclinical studies have shown its anti-proliferative and pro-apoptotic effects, both in short-term treatments targeting TERT’s extratelomeric functions and in long-term treatments causing telomere attrition [78,91,95,129]. However, despite the promising preclinical results, even in the context of B-cell malignancies (Table 1), BIBR’s poor pharmacokinetic properties have hindered its clinical development. To address this limitation, nanotechnology-based drug delivery systems such as zeolitic imidazole framework-8 (ZIF-8) have been explored. ZIF-8 enhances the nuclear transport and release of BIBR1532, improving TERT activity inhibition and anti-cancer effects [130].

Natural compounds have also emerged as telomerase inhibitors. For instance, the epigallocatechin derivative MST-312, similar to BIBR1532, has been shown to disturb the TEN domain conformation of TERT, thus inhibiting telomerase activity [112]. MST-312 induces acute growth arrest and apoptosis in cancer cells during short-term treatment [99]. Notably, treatment with MST-312 after stem cell transplantation in multiple myeloma patients resulted in a slight, but not significant, reduction in the stem cell survival rate, accompanied by significantly improved progression-free survival compared to the untreated group [131].

Indirect telomerase inhibitors include G-quadruplex stabilizers, such as telomestatin, BRACO-19, and CX-5461 (Pidnarulex), which stabilize guanine-rich DNA structures at telomeres, thereby blocking telomerase and inducing DNA damage, leading to cell death [132,133,134].

Nucleoside analogs, such as 6-thio-deoxyguanosine, are incorporated into de novo-synthesized telomeres by telomerase, leading to telomere dysfunction. By mimicking uncapped telomeres, these analogs trigger the DDR and induce apoptosis specifically in telomerase-positive cells. While promising in preclinical studies, these compounds remain in the early development stages [135].

An emerging and promising strategy for cancer treatment is suicide gene therapies, particularly through oncolytic virotherapy. One prominent example is Telomelysin (OBP-301), a replication-competent oncolytic adenovirus engineered with a *TERT* promoter, enabling selective replication in telomerase-positive cancer cells [136,137].

TERT-targeted therapeutic vaccines are designed to activate T cells that recognize specific tumor antigens, thus enhancing the immune response against cancer cells. Peptide vaccines such as GV1001, GX301, UV1, and Vx-001, which consist of short amino acid chains derived from the full-length TERT sequence, along with DNA vaccines like INVAC-1, a DNA plasmid encoding a modified TERT protein, have undergone extensive clinical evaluation. Studies have shown that these vaccines are generally safe and well tolerated while providing survival benefits [138,139]. Notably, INVAC-1 has also been investigated in CLL (NCT03265717), although no clinical data from this trial have been reported to date.

Adoptive T-cell therapies, such as T cells engineered with an HLA-A2-restricted T-cell receptor (TCR) that recognizes human TERT with high affinity, represent an emerging strategy [138]. Notably, TERT-specific TCR-engineered T cells have shown efficacy in suppressing human CLL progression in humanized mice [140].

It is important to note that most of these approaches primarily aim to inhibit TERT’s function in telomere elongation. Targeting the extratelomeric functions of TERT represents a promising alternative or, more compellingly, a complementary strategy to telomere-directed therapy. This approach could simultaneously target multiple cancer hallmarks rather than exclusively addressing replicative immortality. Among the strategies discussed, only BIBR1532 and MST-312 have demonstrated effects on the non-canonical functions of TERT, likely due to their mechanisms of action, which involve direct binding to the TERT subunit. However, they remain unsuitable for clinical application. Additional research is essential to uncover innovative drugs and strategies that leverage TERT’s extratelomeric functions beyond telomere maintenance.

### Importance of TERT Inhibition in B-Cell Malignancies

Inhibiting telomerase could effectively limit cancer cell proliferation, ultimately leading to senescence and apoptosis. However, strategies focusing solely on inhibiting the pivotal role of telomerase may face challenges, such as the prolonged treatment durations required for significant telomere shortening and dysfunction and the risk of toxicity to normal tissues [122]. This aspect is particularly relevant in B-cell malignancies, both those driven by EBV and those unrelated to viral infections, as variations in the telomere length may influence the efficacy of therapeutic approaches targeting only this canonical function of telomerase.

In this context, the evidence of TERT’s extratelomeric functions in regulating proliferation and viability pathways opens up new therapeutic strategies. Targeting TERT’s non-canonical functions could achieve anti-cancer effects independently of the telomere lengths of tumor cells. Moreover, the observation that TERT inhibition per se induces pro-apoptotic effects and sensitizes tumor cells to chemotherapeutic agents is promising. For example, combined treatments using TERT inhibitors, such as BIBR1532 and MST-312, with drugs used in B-cell malignancies, such as Cyclophosphamide, Fludarabine, or Doxorubicin, have demonstrated promising results in both in vitro and in vivo models [78,91,95,96,100]. This highlights short-term TERT inhibition as an innovative complementary strategy to enhance existing therapies. Moreover, further studies exploring synthetic lethality interactions between TERT’s extratelomeric functions and genetic alterations in B-cell malignancies could provide a rationale for combination treatments involving TERT inhibitors and targeted therapies, ultimately contributing to the optimization of treatment strategies.

It should be noted that, due to its extratelomeric functions, TERT represents a promising therapeutic target at all disease stages, as its inhibition restricts the proliferative ability and induces apoptosis regardless of the tumor cell telomere length, while synergizing with standard chemotherapy. This is even more relevant in relapsed or therapy-resistant B-cell neoplasms, where intrinsic (microenvironmental) and extrinsic (therapeutic) pressures drive the emergence of subclones with the activation of multiple cancer-related pathways, contributing to disease refractoriness and tumor progression [141]. In this context, targeting TERT’s extratelomeric functions could effectively suppress tumor proliferation and promote apoptosis.

## 7. Conclusions and Future Perspectives

In B-cell malignancy models, strategies aimed at inhibiting TERT have shown that short-term treatments can strongly impair tumor cell proliferation and viability through mechanisms largely independent of the telomere length, as they are obtained within timeframes shorter than those required for telomere attrition and dysfunction (Table 1).

These effects involve complex regulatory loops with key signaling pathways, such as NF-κB, AKT, and MYC, which are critical for tumor B-cell survival and proliferation (Figure 1). While mechanistic insights have clarified some extratelomeric functions of TERT, particularly its role in gene expression regulation, many aspects remain unexplored. In particular, its involvement in some signaling cascades and its role in mitochondrial protection and oxidative stress regulation warrant further investigation.

An important open issue is that, while telomerase is inactive in most somatic cells, it remains active in certain stem cell populations [142]. Currently, no data are available on the extratelomeric functions of TERT in normal stem cells. Normal tissue stem cells are telomerase-competent but largely quiescent, requiring only transient activation to support the regenerative potential of high-turnover organs [142]. In contrast, cancer cells exhibit high and sustained telomerase activity, suggesting that high TERT levels may drive non-canonical functions specifically in cancer cells.

To fully elucidate the diverse roles of TERT beyond telomere maintenance, it is essential to develop cellular and animal models with tunable TERT expression. These models would enable the deeper exploration of extratelomeric functions in relation to different expression levels and activation timings.

Addressing these questions will not only deepen our understanding of the biological processes driving cancer progression but also highlight the therapeutic potential of TERT beyond telomere length regulation. The growing understanding of TERT’s non-canonical roles in sustaining B-cell malignancies suggests that short-term TERT inhibition could prevent tumor growth and enhance the sensitivity to existing treatments, offering a promising strategy to improve cancer therapies while minimizing the toxicity to normal tissues. Further elucidation of the precise mechanisms underlying TERT’s extratelomeric functions will identify novel therapeutic agents and optimize treatment strategies, ultimately leading to more effective and specific cancer therapies.

## Figures and Tables

**Table 1:** TERT’s non-canonical functions in models of B-cell malignancies.

B-Malignancy	TERT’s Extratelomeric Function(s)	Reference
EBV-immortalized lymphoblastoma cell lines	Increased TERT levels promote EBV latency program, increase resistance to lytic cycle induction, and enhance in vitro growth properties.	[68]
EBV-immortalized lymphoblastoma cell lines; EBV-negative and -positive BL cell lines	TERT inhibition via shTERT RNA, decreasing BATF and increasing *BZLF1* expression, induces the EBV lytic cycle. In both EBV-positive and -negative cells, TERT inhibition reduces proliferation and triggers AKT1/FOXO3/NOXA-dependent apoptosis.	[89]
EBV-immortalized lymphoblastoma cell lines	High TERT levels activate NOTCH2 through the NF-κB signaling pathway. In turn, NOTCH2 induces BATF, which suppresses *BZLF1* viral expression, thereby promoting EBV latency.	[90]
EBV-immortalized lymphoblastoma cell lines; EBV-positive and -negative BL cell lines	Short-term inhibition of TERT by BIBR1532 causes cell cycle arrest and apoptosis, associated with activation of DDR independently of telomere shortening. TERT inhibition sensitizes cells to the pro-apoptotic effects of chemotherapeutic agents.	[91]
EBV-immortalized lymphoblastoma cell lines; EBV-negative BL cell lines	Short-term TERT inhibition by BIBR1532 reduces proliferation and impairs the viability of LCL and BL cells xenografted in zebrafish through cell cycle arrest and apoptosis driven by DDR activation, independently of telomere shortening.	[92]
EBV-immortalized lymphoblastoma cell lines; EBV-negative BL cell lines	Short-term inhibition of TERT by BIBR1532 impairs cell growth by downregulating MYC via NF-κB signaling. Combined treatment with TERT inhibitor and chemotherapeutic agents induces a cumulative inhibitory effect on the proliferation of LCL and BL cells xenografted in zebrafish.	[78]
EBV-positive B-cell lymphoma cell line	Triptolide inhibits TERT expression and activity by downregulating SP1 and MYC. Triptolide promotes the lytic cycle of EBV.	[93]
Lymphoblastoma cell line and primary leukemic cells from B-ALL	Short-term inhibition of TERT by shTERT RNA decreases MYC protein stability, leading to the reduced transcription of its target genes and impairing cell viability without affecting the telomere length.	[77]
Primary leukemic cells from ALL	Telomerase inhibition with MST-312 for 48 hours significantly reduces levels of *IL6* in primary leukemic cells by inhibiting NF-κB signaling.	[76]
Pre-B ALL cell line	Treatment with BIBR1532 for 48 h impairs cell proliferation and causes cell death, likely by reducing Survivin-mediated *MYC* and *TERT* levels.	[94]
Pre-B ALL cell line	Treatment with BIBR1532 for 24 h enhances ROS production and increases the anti-proliferative and pro-apoptotic effects of doxorubicin by upregulating *TP73* and *p21* and downregulating *MYC* and *TERT*.	[95]
Pre-B ALL cell lines	The telomerase inhibitor MST-312 shows dose-dependent cytotoxic and apoptotic effects on pre-B ALL cells. A 48 h combination with doxorubicin enhances cytotoxicity and apoptosis, linked to reductions in *BCL2*, *MYC*, and *TERT* and an increase in *BAX*.	[96]
Pre-B ALL cell line	A 48 h combined treatment with MST-312 and NU7441, a DNA-PK inhibitor, synergistically induces anti-proliferative and pro-apoptotic effects, downregulating *MYC*, *TERT*, and *BCL2* and upregulating *BAX*.	[97]
Pre-B ALL cell lines	Short-term TERT inhibition by siRNA reduces proliferation and viability, associated with the upregulation of *BAX* and downregulation of *BCL2*. The cytotoxicity of TERT inhibition is characterized by the upregulation of ferroptosis promoters (lipid-ROS, *ACSL4*) and suppression of inhibitors (*SLC7A11*).	[98]
Human multiple myeloma cell line	Treatment with MST-312 for 48 h induces anti-proliferative and pro-apoptotic effects, by downregulating *MYC*, *TERT*, *BCL2*, *IL6*, and *TNFα* and upregulating *BAX*.	[99]
Multiple myeloma cell lines	Treatment with BIBR1532 for 48 h inhibits cell proliferation and promotes apoptosis associated with the downregulation of TERT, MYC, BCL-XL, and Survivin; increased BAD levels; the dephosphorylation of PI3K, AKT1, and mTOR; and the increased phosphorylation of ERK1/2 and MAPK. BIBR1532, combined with doxorubicin or bortezomib, exhibits a synergistic pro-apoptotic effect.	[100]
Multiple myeloma cancer stem cells from cell lines and primary clinical specimens	Short-term telomerase inhibition by Imetelstat reduces clonogenic growth and promotes differentiation by downregulating stemness-related genes, without affecting the telomere length.	[101]
Philadelphia chromosome-positive B-lymphoblastic leukemia cell line	Short-term treatment with the telomerase inhibitor Imetelstat demonstrates dose-dependent suppression of cell proliferation, unrelated to telomere length: at higher concentrations, it induces increased levels of γH2AX.	[102]

TERT: telomerase reverse transcriptase; EBV: Epstein–Barr virus; LCL: EBV-immortalized lymphoblastoma cell lines; BL: Burkitt lymphoma; shTERT: short-hairpin RNA against TERT; BATF: basic leucine zipper ATF-like transcription factor; BZLF1: BamHI Z fragment leftward open reading frame 1; AKT1: AKT serine/threonine kinase 1; FOXO3: forkhead box O3; NOXA: phorbol-12-myristate-13-acetate-induced protein 1; NOTCH2: notch receptor 2; NF-κB: RELA proto-oncogene, NF-kB subunit; DDR: DNA damage response; MYC: MYC proto-oncogene, bHLH transcription factor; SP1: Sp1 transcription factor; ALL: acute lymphocytic leukemia; IL6: interleukin 6; h: hours; Survivin: baculoviral IAP repeat containing 5, BIRC5; ROS: reactive oxygen species; TP73: tumor protein p73; p21: cyclin-dependent kinase inhibitor 1A, CDKN1A; BCL2: BCL2 apoptosis regulator; BAX: BCL2-associated X, apoptosis regulator; DNA-PK: DNA-dependent protein kinase; ACSL4: acyl-CoA synthetase long-chain family member 4; SLC7A11: solute carrier family 7 member 11, cystine/glutamate transporter; TNFα: tumor necrosis factor; BCL-XL: BCL2-like 1, BCL2L1; PI3K: phosphatidylinositol-4,5-bisphosphate 3-kinase catalytic subunit alpha, PIK3CA; mTOR: mammalian target of rapamycin; ERK1/2: extracellular signal-regulated kinase 1/2; MAPK: mitogen-activated protein kinase 1; γH2AX: phosphorylated H2A.X variant histone.

## Abbreviations

The following abbreviations are used in this manuscript:

TERT	telomerase reverse transcriptase
TERC	telomerase RNA component
DDR	DNA damage response
ATM	ATM serine/threonine kinase
ATR	ATR serine/threonine kinase
TP53	tumor protein p53
RB1	RB transcriptional corepressor 1
p16	cyclin-dependent kinase inhibitor 2A
ALT	Alternative Lengthening of Telomeres
SP1	Sp1 transcription factor
MYC	MYC proto-oncogene, bHLH transcription factor
HIF1A	hypoxia-inducible factor 1 subunit alpha
AP-2	transcription factor AP-2 alpha
ETS	E-twenty-six
TCF	ternary complex factors
NF-κB	RELA proto-oncogene
β-catenin	catenin beta 1
WT1	Wilms’ Tumor 1
NFX-1	Nuclear Transcription Factor X-Box Binding
MAD1	MAX dimerization protein 1
CTCF	CCCTC Binding Factor
PI3K	phosphatidylinositol-4,5-bisphosphate 3-kinase catalytic subunit alpha
AKT1	serine/threonine kinase 1
HBV	hepatitis B virus
HCV	hepatitis C virus
KSHV	Kaposi’s sarcoma-associated herpes virus
EBV	Epstein–Barr virus
CMV	cytomegalovirus
HTLV-1	human T-cell leukemia virus-1
LCL	lymphoblastoid cell line
LMP1	latent membrane protein 1
MAPK	mitogen-activated protein kinase 1
ERK1/2	extracellular signal-regulated kinase 1/2
GC	germinal center
DLBCL	diffuse large B-cell lymphoma
BL	Burkitt lymphoma
CLL	chronic lymphocytic leukemia
WNT	wingless-type MMTV integration site family
BCL2	BCL2 apoptosis regulator
P21	cyclin-dependent kinase inhibitor 1A
FOXO3	forkhead box O3
NOXA	phorbol-12-myristate-13-acetate-induced protein 1
BAD	BCL2-associated agonist of cell death
NOTCH2	notch receptor 2
BATF	basic leucine zipper ATF-like transcription factor
BZLF1	BamHI Z fragment leftward open reading frame 1
ZIF-8	zeolitic imidazole framework-8
ROS	reactive oxygen species
BRG1	SWI/SNF-related BAF chromatin remodeling complex subunit ATPase 4
IL6	interleukin 6
TNFα	tumor necrosis factor
NME2	NME/NM23 nucleoside diphosphate kinase 2
TOM 20	translocase of outer mitochondrial membrane 20
TOM 40	translocase of outer mitochondrial membrane 40
TIM 23	translocase of inner mitochondrial membrane 23
COX	cyclooxygenase-1
ACSL4	acyl-CoA synthetase long-chain family member 4
SLC7A11	solute carrier family 7 member 11
